# An Academic Genealogy of Psychometric Society Presidents

**DOI:** 10.1007/s11336-018-09651-4

**Published:** 2019-01-17

**Authors:** Lisa D. Wijsen, Denny Borsboom, Tiago Cabaço, Willem J. Heiser

**Affiliations:** 1 Department of Psychological Methods, University of Amsterdam, Nieuwe Achtergracht 129-B, 1018 WS Amsterdam, The Netherlands; 2 Department of Psychology, Humboldt University, Berlin, Germany; 3 Department of Psychology, Leiden University, Leiden, The Netherlands

**Keywords:** history of psychometrics, academic genealogy, genealogical trees

## Abstract

In this paper, we present the academic genealogy of presidents of the *Psychometric Society* by constructing a genealogical tree, in which Ph.D. students are encoded as descendants of their advisors. Results show that most of the presidents belong to five distinct lineages that can be traced to Wilhelm Wundt, James Angell, William James, Albert Michotte or Carl Friedrich Gauss. Important psychometricians Lee Cronbach and Charles Spearman play only a marginal role. The genealogy systematizes important historical knowledge that can be used to inform studies on the history of psychometrics and exposes the rich and multidisciplinary background of the Psychometric Society.

**Electronic supplementary material:**

The online version of this article (10.1007/s11336-018-09651-4) contains supplementary material, which is available to authorized users.

## Introduction

Psychometrics is a scientific discipline concerned with ‘quantitative measurement practices in psychology, education and the social sciences’ (‘What is psychometrics?’, 2018). The origin of psychometrics is often traced to the primary example of a model for measurement: the common factor model, constructed by Charles Spearman (1904), in which a set of observed variables is regressed on a common latent variable. This model provided the classical decomposition of test scores into general and item-specific sources of variance that enabled psychologists and psychometricians to systematically measure what Spearman called *general intelligence* or *g*. Psychometric research, commonly understood to cover the technical rather than the substantive side of test theory (Borsboom, 2006), has further developed and branched out in a wide array of modeling techniques such as classical test theory (CTT), structural equation modeling (SEM), item response theory (IRT), and multidimensional scaling (MDS) (Jones & Thissen, 2007).

Variations of these models have been used for the measurement and prediction of a multitude of psychological attributes, such as personality dimensions (McCrae & Costa, 1987), mental abilities (Thurstone, 1938; Carroll, 1993), and psychiatric disorders (Caspi et al., 2014). In addition, implementations of psychometric testing have resulted in a massive set of practical test applications; examples include college admission tests (e.g., the SAT’s or the Medical College Admission Test), various psychological assessments for job performance (Bowling & Hammond, 2008), and clinical assessments used in psychiatric practice (Floyd & Widaman, 1995). As a result, at the beginning of the twenty-first century, circumventing the psychological test has become almost impossible in large parts of the world.

Even though psychometrics has thus taken up a prominent position in the scientific domain, and in society at large, little research has been done on its origins and its development through time (exceptions are: Groenen & van der Ark, 2006; Jones & Thissen, 2007; Van der Heijden & Sijtsma, 1996). As a result, we have a limited view of the lines of intellectual descent that have led to the current structure of the psychometric field. This article aims to contribute to a better understanding of these issues by providing an academic genealogy of Psychometric Society presidents. An academic genealogy is a genealogical tree, in which the traditional ancestor–descendent relations are replaced by relations between doctoral advisors and students (Kealy & Mullen, 1996). As such, the academic genealogy provides an overview of one or more scientific disciplines through a simple but clear visualization of advisor–student relations.

In historical research, academic genealogies have been used to trace the descent of one single person (Bennet & Lowe, 2005; Williams, 1993), to identify causes of unresolved disagreements in medical science (Hirshman et al., 2016), or for tracing scientific ideologies back to their roots (Robertson, 1994). The use of genealogical trees has also proven popular outside of professional historical research, as can be seen from the fact that several websites have been developed for this purpose (e.g., *PhDTree, the Mathematics Genealogy Project*, *Neurotree*). Finally, throughout the sciences there is a general trend of constructing academic genealogies to uncover the evolution of individual disciplines, such as mathematics (Gargiulo, Caen, Lambiotte & Carletti, 2016), astronomy (Tenn, 2016), biology (Bennett & Lowe, 2005), psychology (Williams, 1993; Robertson, 1994), and primatology (Kelley & Sussman, 2007). In the current study, we aim to contribute to this development by constructing a well-documented and verifiable academic genealogy of Psychometric Society presidents that can function as a resource for historians of psychometrics and as such may help to yield insights into the questions of how psychometrics originated as a new scientific discipline and how it has developed over time.

The structure of this paper is as follows. The methods section will elaborate on the methods used for the collection of the data and the construction of the genealogies. In the results section, five distinct genealogies are presented, each accounting for a number of presidents. In the discussion section, we will provide some ideas for further historical research and discuss some limitations.

## Methods

To minimize selection bias while keeping the practical task within reasonable bounds, we have chosen to construct a genealogy in which presidents of the Psychometric Society function as the initial set, from which we work ‘backwards in time’ to uncover each of their lineages. There are several reasons for selecting the presidents of the Psychometric Society as our starting point. The Psychometric Society, originally founded in 1935 by L.L. Thurstone and others, is the most important institution concerned with psychometric science, and Psychometrika (founded by Paul Horst in 1936) its flagship journal (Heiser & Hubert, 2016). By organizing the most central meeting of psychometricians—the International Meeting of the Psychometric Society (IMPS)—the Psychometric Society is the main social structure in the psychometric community. As a result, presidents of the Psychometric Society, elected by the members of the Psychometric Society, are invariably central figures in the discipline, with important social and scientific functions in psychometrics. Therefore their genealogy is both intrinsically interesting and likely to contain important historical information. Of course, that presidents are typically important psychometricians does not imply that psychometricians who did not become president are unimportant, and the genealogy covers only the history of the Psychometric Society. However, given the requirement of the current study, that each link in the tree must be backed by historical evidence, practical constraints necessitate that we leave extending the tree beyond this set to a future study.

Up until 2017, the Psychometric Society has counted 84 presidents, including the president-elect, all of whom have taken up prominent positions in the field of psychometric research in their time. By taking these 84 presidents as our initial set of nodes, the genealogy was constructed in an objective, systematic fashion: each individual advisor–student relation has been assessed through careful archival research and is adequately documented. However, it is important to note that, because the initial set in the tree represents the presidents of the Psychometric Society, other nodes include only those scholars that are necessary for connecting these presidents back to their ancestors. This means that nodes were added only for (a) representing a president directly or (b) tracing the line of descent of a president back through time. Once different lineages shared a common ancestor in the nineteenth century, further investigation of the line of descent was stopped. This both served to limit the number of nodes added to the tree and to ensure that the genealogical tree would not stretch out over too long a period of time. When an individual lineage did not share any common ancestors with any other lineage, we aimed to trace the lineage back into the nineteenth century.

To collect all the necessary information on student–advisor relations, several archival sources were consulted. These included doctoral dissertations, obituaries, monographs, resumes, university websites and the *Mathematics Genealogy Project*. Online academic genealogies that are open for edits by users, such as *Neurotree* (David & Hayden, 2012) or *PhDTree* (now taken offline), proved to be helpful sources as well, though connections taken from these websites were always double checked for accuracy. When online resources were not conclusive, we also engaged in personal communication with presidents or with archivists of university libraries. In the data collection phase, we coded each person’s supervisor, their year of graduation and the university where they wrote their dissertation.

To ensure a systematic approach, we applied a number of rules. Firstly, we have only included relations that are of the advisor–graduate student kind and thus excluded any other influential relations. Secondly, we have only included one advisor per person. Thirdly, we only included advisors of people who actually finished their dissertation and got an official PhD degree. If people do not have a PhD degree, they are simply the start of the lineage.

## Results

Following the rules, we set in the methodology section, the total academic genealogy of psychometrics includes 208 scholars, of which 84 are presidents. This genealogy comprises 5 separate genealogies, leading back to James R. Angell, Wilhelm Wundt, William James, Carl Friedrich Gauss or Albert Michotte. Sixty-four out of 84 presidents connect to one of these genealogies.

Sixty-six out of 84 of the presidents received their PhD degree in the USA, 14 in Europe, 1 in Asia, 1 in Africa and 1 in Australia. Five presidents are females and represent the only women in this genealogy. Universities that have produced a large number of presidents are the University of Chicago ($n = 14$), Princeton University ($n = 9$), Columbia University ($n = 8$), the University of Illinois ($n = 6$) and Stanford University ($n = 5$).

**Fig. 1:**
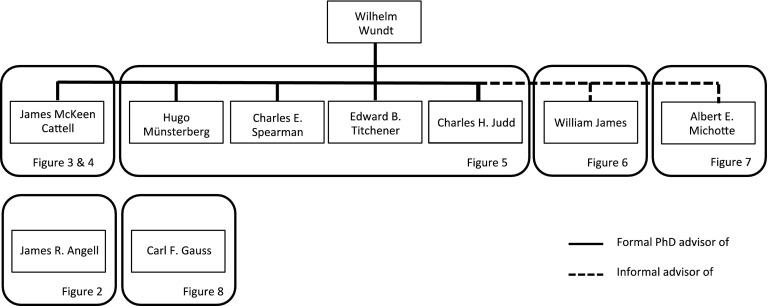
Overview of the different genealogies in this paper. The genealogies of Cattell, Münsterberg, Spearman, Titchener and Judd, doctoral students of Wundt, will be discussed in Figs. 3, 4 and 5. Figures 6 and 7 show the genealogies of James and Michotte (non-doctoral students of Wundt), and the genealogies of Angell and Gauss in Figs. 2 and 8.

**Fig. 2:**
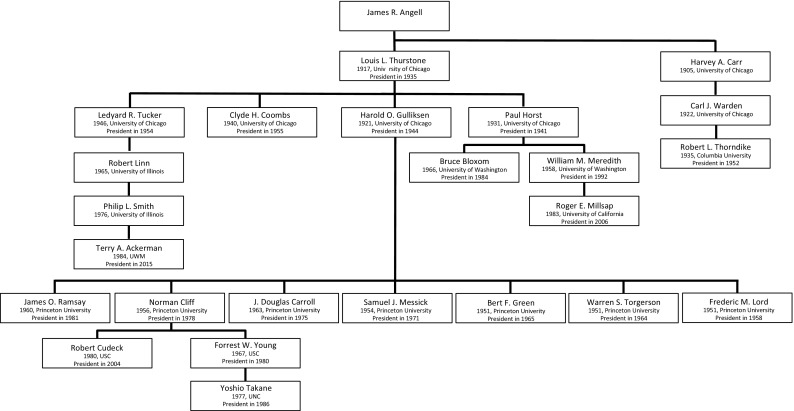
The genealogy of James R. Angell. For each scholar in the genealogy, the year of obtaining a PhD Degree and the university in question, are given. For presidents of the Psychometric Society, also the year of their presidency is included.

**Fig. 3:**
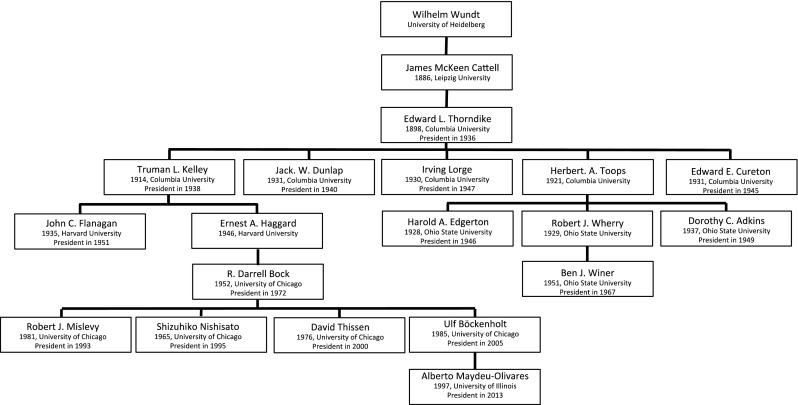
The Genealogy of Wilhelm Wundt, part I. For each person in the genealogy, the year of obtaining a PhD Degree and the university in question are given. For presidents of the Psychometric Society, also the year of their presidency is included.

**Fig. 4:**
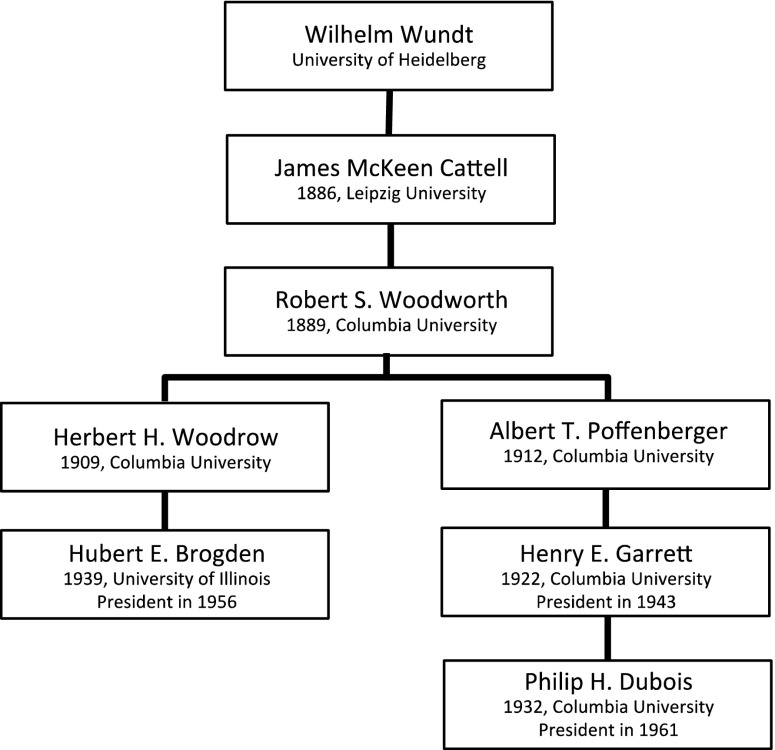
Genealogy of Wilhelm Wundt, part II. For each scholar in the genealogy, the year of obtaining a PhD Degree and the university in question, are given. For presidents of the Psychometric Society, also the year of their presidency is included.

**Fig. 5:**
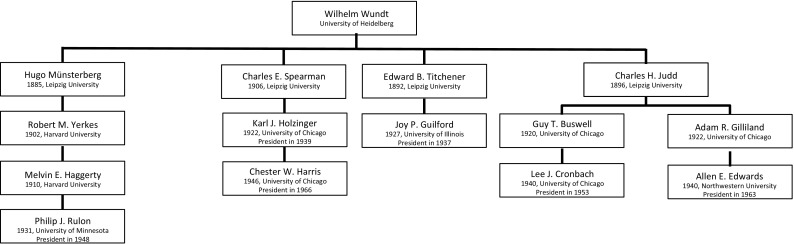
Genealogy of Wilhelm Wundt, part III. For each scholar in the genealogy, the year of obtaining a PhD Degree and the university in question are given. For presidents of the Psychometric Society, also the year of their presidency is included.

**Fig. 6:**
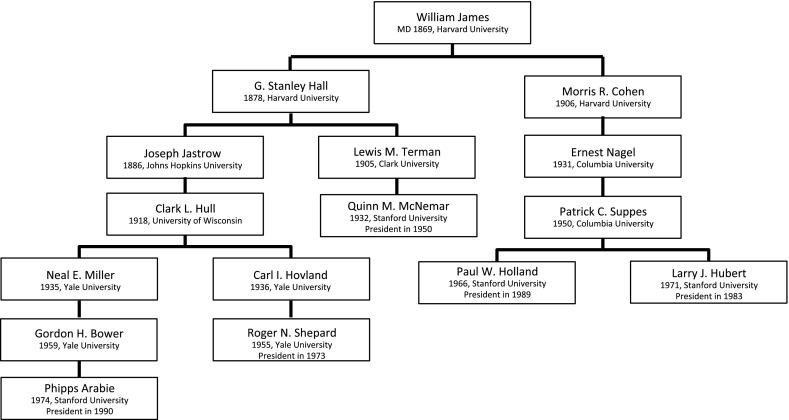
The genealogy of William James. For each scholar in the genealogy, the year of obtaining a PhD Degree and the university in question are given. For presidents of the Psychometric Society, also the year of their presidency is included.

**Fig. 7:**
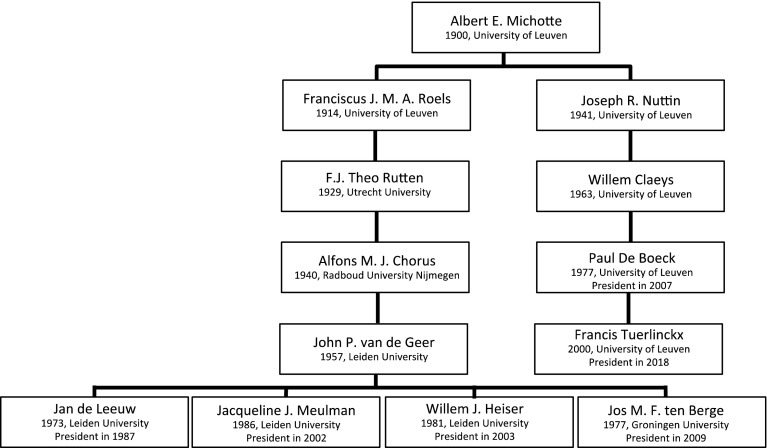
The genealogy of Albert E. Michotte. For each scholar in the genealogy, the year of obtaining a PhD Degree and the university in question, are given. For presidents of the Psychometric Society, also the year of their presidency is included.

**Fig. 8:**
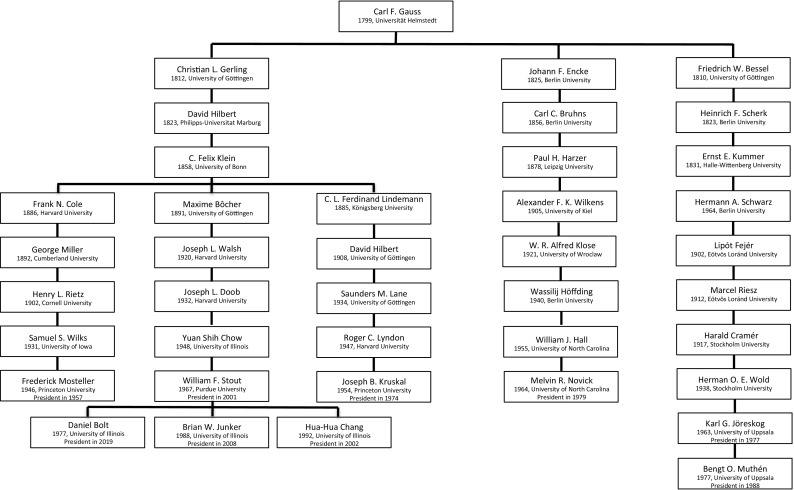
The genealogy of Carl Friedrich Gauss. For each scholar in the genealogy, the year of obtaining a PhD Degree and the university in question are given. For presidents of the Psychometric Society, also the year of their presidency is included.

Figure 1 provides an overview of the five different genealogies, which are visualized in Figs. 2, 3, 4, 5, 6, 7 and 8. Figures 3, 4 and 5 show the genealogies of the official doctoral students of Wilhelm Wundt. Due to James McKeen Cattell’s sizeable lineage, it has been divided over two figures. The genealogies of James R. Angell (Fig. 2), William James (Fig. 6), Albert E. Michotte (Fig. 7) and Carl Friedrich Gauss (Fig. 8) have no official connections to Wilhelm Wundt and are given separately. Table 1 provides the lineages of the presidents that are not part of any of the larger genealogies. Tables 2 to 5 (see the Supplementary Material) provide the references of the sources we used for each advisor–student relation in this genealogy.^1^

### The Genealogy of James R. Angell

The first genealogy we discuss starts with James R. Angell, an American psychologist who never officially finished his dissertation and is hence the starting point of this tree (see Fig. 2). Angell’s genealogy holds 25 scholars of which 20 are presidents of the Psychometric Society. One of Angell’s major accomplishments was his presidency of Yale University from 1921 until 1937. One of Angell’s students was Louis L. Thurstone, the founder and first president of the Psychometric Society. Thurstone became very well known for his work on scaling (Thurstone, 1927) and multiple factor analysis (Thurstone, 1935). Four of the psychometricians who descended from Thurstone were each president of the Psychometric Society in its early days: Ledyard R Tucker, Clyde Coombs, Harold Gulliksen, and Paul Horst.

Paul Horst, president in 1941 and 1942, was one of the six founders of Psychometrika and successfully pursued the idea of setting up a journal dedicated to mathematically oriented psychological research (Heiser & Hubert, 2016). Many others supported him in this endeavor, among whom future presidents Jack Dunlap (see Fig. 3), Joy P. Guilford (see Fig. 5) and Harold Gulliksen (see Fig. 2). Years later, two of Horst’s students became presidents of the Psychometric Society: William Meredith, in 1992, and Bruce Bloxom, in 1984 (see Fig. 2). Meredith was the advisor of Roger E. Millsap, who shared with him an interest in measurement invariance and who became president in 2006.

Ledyard R Tucker, president in 1954, wrote his dissertation in 1941. In his early career, he worked on factor analysis together with Thurstone. From 1944 until 1960, he became the first director of statistical analysis at the Educational Testing Service (ETS) and worked as a lecturer at Princeton University. After that he became professor of psychology at University of Illinois. As Bert F. Green (1980) eloquently puts it, Ledyard Tucker had a lifelong ‘affair with psychometrics’. Tucker’s lineage goes to Robert Linn, educational psychologist, to Philip L. Smith, to Terry Ackerman, who was president in 2015. Ackerman now holds the ‘Lindquist Chair’ at ACT.

Harold Gulliksen, president in 1944, turns out to be one of the most fruitful suppliers of presidents of the Psychometric Society in the entire genealogy: seven presidents were directly advised by Gulliksen, and another three are of the next two generations, advised by either Norman Cliff or Forrest W. Young (see Fig. 2). During the Second World War, Gulliksen worked for the navy, where he introduced many different selection and assessment instruments. He noted that assessment instruments should not only include tasks that reflect skills they learn during the training, but also skills they actually employ on the job (Messick, 1998). Gulliksen’s *Theory of Mental Tests* (1950) was one of the first comprehensive handbooks on classical test theory. In 1945, he was appointed professor of psychology at Princeton University until 1972 and also worked at ETS after WWII as a research advisor until 1974, doing both half time. Besides an interest in mental testing, Gulliksen also had a strong interest in learning and used mathematical models to visualize learning curves (Messick, 1998).

Another president and student of Thurstone who was well known for his interest in mathematical psychology was Clyde Coombs, president in 1955. Throughout his career, he published on analysis of qualitative structures of proximity and dominance data (Coombs, 1952, 1964) and models of conflict and choice (Tversky, 1992).

A surprising presence in Angell’s genealogy is Robert L. Thorndike, the son of Edward L. Thorndike [who is part of Wundt’s genealogy (Fig. 3), not Angell’s]. After writing a dissertation on animal learning, he specialized in educational psychology and made contributions to the field of reliability. Like his father, he stayed at Columbia University throughout his entire career and worked closely together with Irving Lorge (Fig. 3). He was president in 1952.

#### Harold Gulliksen’s Graduate Students

As mentioned earlier, Gulliksen was extremely productive when it came to preparing his students for the presidency of the Psychometric Society: no less than seven of his students eventually became president of the Psychometric Society. The first three to write their dissertations, in 1951, were Warren S. Torgerson, Bert F. Green and Frederic M. Lord.

In his dissertation, Torgerson developed a method of multidimensional scaling (MDS), a new research area that gained a prominent presence in psychometric research. After finishing his degree, he spent some time at the Navy, where he headed the statistical analysis division. In 1955, he returned to Princeton University and resumed his work on MDS (Green, 1999). Bert F. Green was president in 1965, one year after Torgerson’s presidency in 1964, and was editor of *Psychometrika *between 1972 and 1980.

Frederic M. Lord, president in 1958, was probably one of the most famous psychometricians that ever lived. Together with Melvin Novick (see Fig. 8), he wrote perhaps the most significant textbook in the history of psychometrics: *Statistical Theories of Mental Test Scores* (1968). In this book, Lord and Novick provided a mathematical account of classical test theory and an introduction into modern test theory. From 1949 onwards, Lord worked at ETS as Director of Statistical Analysis and wrote his dissertation at Princeton in 1951. During this time, he became an important proponent of Item Response Theory (IRT).

J. Douglas Carroll wrote his dissertation in 1963, and left for Bell Labs as Member of Technical Staff after finishing his dissertation (Heiser, 2013). From 1925 until 1996, Bell Labs was the research center of AT&T, where many scientists of a wide variety of disciplines worked side by side on new ideas for information technology. Carroll’s dissertation still focused on human learning, but under the influence of many fellow psychometricians who also worked at Bell Labs (among others Roger Shepard, Fig. 6, and Joe Kruskal, Fig. 8) he made the switch to MDS. Over the years, Carroll invited many other psychometricians to spend some time at Bell Labs, and Bell Labs became the most important center for MDS-related research.

Samuel Messick wrote his dissertation in 1954, after which he joined ETS in 1956, where he stayed until he retired as vice-president in 1997. One of his main interests was the concept of validity, in which he stressed the idea that validity was not purely a property of a test, but also a property of the interpretation the test scores (Messick, 1989). Norman Cliff wrote his dissertation in 1957, succeeded Bert Green as editor of *Psychometrika* (1981–1984), and supervised two presidents of the Psychometric Society: Robert Cudeck, professor in quantitative psychology at Ohio State University, and Forrest W. Young. Forrest Young was professor in quantitative psychology at the University of North Carolina and was the advisor of Yoshio Takane, president in 1986. James O. Ramsay, famous for his work on functional data analysis, was the last president of the Psychometric Society to write his dissertation under Gulliksen’s supervision.

### The Genealogy of Wilhelm Wundt

The largest genealogy starts with Wilhelm Wundt. Wundt’s genealogy counts 40 scholars, of whom 24 are presidents. Due to its scope, the entire Wundt genealogy is divided over three graphics: Figs. 3, 4 and 5.

The German Wilhelm Wundt is often seen as the founder of experimental psychology and led the first psychology laboratory in Leipzig. Wundt oversaw an exceptional number of 184 doctoral dissertations between 1875 and 1919, and many became famous psychologists or psychometricians, five of whom can be found in this genealogy: James McKeen Cattell (Figs. 3 and 4), Hugo Münsterberg, Charles Spearman, Charles H. Judd and Edward Titchener (Fig. 5). Wundt’s influence was not limited to Europe; he also attracted many American psychologists, like Cattell and Titchener, who travelled all the way to Europe to spend time at Wundt’s laboratory. Inspired by physiological experiments, Wundt initiated a line of research in which psychological objects, such as sensory stimuli, were investigated through experiments (Danziger, 1994). Wundt believed that experiments were only suitable for direct responses to physical stimuli that are bereft of any interpretation. The participants in his experiments were his own students, and they were thoroughly trained in how to accurately report their responses. Important to note here is that Wundt’s psychology was not limited to experiments. He found experimental psychology the most suitable method for discovering psychological laws, but he believed that psychological experiments should not take up the entire space of psychological science. He considered topics like language, myth, culture and religion not suitable for experimental psychology and used *Völkerpsychologie* as a method for investigating those (Danziger, 1994).

Though the five direct descendants of Wundt wrote their dissertations in Germany, all of the following generations wrote their dissertations in the USA, predominantly at Columbia University, Harvard University, the University of Chicago and the University of Illinois. At the time, the USA, and especially the University of Chicago, had become the center of psychometrics with Louis L. Thurstone steering the wheel (see Fig. 2).

James McKeen Cattell (Figs. 3 and 4) was the first American to obtain a PhD degree in psychology. His work on intelligence testing was strongly influenced by the versatile Francis Galton, who believed that traits were both heritable and measurable. Cattell finished his dissertation with Wundt in 1886, and in 1891, he accepted a professor position at Columbia University (where he was dismissed after speaking up against the World War I draft). This genealogy holds two of his graduate students: Edward L. Thorndike (Fig. 3) and Robert S. Woodworth (Fig. 4).

#### The Genealogy of Edward L. Thorndike

Edward L. Thorndike was a pioneer in learning theory, and among other things, investigated animal learning. Together with Thurstone and J. P. Guilford, Thorndike set up the Psychometric Society and became its second president in 1936. He stayed at Columbia University for his entire career and holds a central position in this genealogy, as the advisor of four presidents: Jack W. Dunlap, Irving Lorge, Truman L. Kelley and Edward E. Cureton. Note that Thorndike’s son, Robert L. Thorndike, was already mentioned in Angell’s genealogy (Fig. 2).

Jack W. Dunlap was one of the driving forces behind the establishment of *Psychometrika* and he was president in 1940. He wrote his dissertation in 1931, which was a human factors study on the design of automobiles, and much of his career centered on industrial psychology. After working at a number of universities, he set up his own company, Dunlap & Associates, which was specialized in industrial consultancy. Irving Lorge, president in 1947, worked closely with E.L. Thorndike, especially in studies on language and communication. Together with Robert L. Thorndike (Fig. 2), he developed the Lorge Thorndike Intelligence Test (1957). Edward E. Cureton, president in 1945, obtained his PhD at the Thorndike laboratory in 1931, on the topic of measurement error. Throughout his career, he contributed to factor analysis and validity (Shrader, 1994).

Truman L. Kelley, president in 1938, was a psychometrician with a strong interest in both statistical methods and educational testing. He wrote books like *Statistical Methods* (1923) and *Fundamentals of Statistics* (1947), but also cooperated with Lewis Terman (see Fig. 6) on the Stanford Achievement Test Battery (1922). When Kelley became professor of psychology at Harvard University in 1931, he became the advisor of John C. Flanagan and Ernest Haggard. John C. Flanagan, president in 1951, became famous for his work on the first aptitude tests for American pilots in the Second World War, which he designed using a technique he called ‘the critical incident technique’ (Flanagan, 1954).

Ernest A. Haggard was the advisor of R. Darrell Bock at the University of Chicago, who wrote his dissertation in 1952. Bock, president in 1972, made significant contributions to multivariate analysis methods and IRT, and was the doctoral advisor of four presidents of the Psychometric Society: David Thissen, Robert Mislevy, Shizuhiko Nishisato and Ulf Böckenholt. The latter was the advisor of Alberto Maydeu-Olivares, president in 2013.

The fourth student of Thorndike in this genealogy was Herbert A. Toops. He worked primarily in the field of industrial psychology and designed tests for mechanical ability for trade schools. At Ohio State University, Toops advised two presidents: Harold A. Edgerton, in 1928, and Dorothy C. Adkins, in 1937. Edgerton, who was president in 1946, was also active in industrial psychology. Adkins was the first female president of the Psychometric Society in 1949. She finished her dissertation in 1937, worked as a test developer between 1940 and 1948, and returned to academia in 1948 at the University of North Carolina where she became professor of psychology (T. G. Thurstone, 1976). She was instrumental in bringing L. L. Thurstone there in 1952, after his retirement at the University of Chicago.

Toops was also the advisor of Robert J. Wherry, an industrial psychologist with strong interests in quantitative methodology. In 1951, he advised Ben J. Winer, who became president in 1967. Winer always worked for a psychology department but had strong interests in statistics, and his *Statistical Principles in Experimental Design* is one of the most cited works in psychological research (Haggbloom et al., 2002).

#### The Genealogy of Robert S. Woodworth

From Cattell we find a second, shorter, lineage that starts with Robert S. Woodworth, who wrote his dissertation under Cattell in 1889. Woodworth and E.L. Thorndike investigated transfer of training; whether or not improvement of one function could also improve another function (Woodworth & Thorndike, 1901). From Woodworth, we find another T-junction: one pathway starts with Albert T. Poffenberger, the other with Herbert Woodrow.

Albert Poffenberger wrote his dissertation in 1912 on physiological psychology and later in his career specialized in applied psychology. In 1922, he advised Henry E. Garrett, who was president of the Psychometric Society in 1943. Henry E. Garrett is most likely the most controversial president on the list. His work mostly dealt with intelligence, especially racial differences in IQ, and he did not shy away from putting his scientific views to political work: Garrett believed that racial differences in intelligence were innate and thus genetic (Garrett, 1961) and that mixing of races would have adverse consequences^2^; as a result, he helped organize a committee of researchers against racial mixing and wrote several papers in defense of racial segregation (Winston, 1998). He was the doctoral advisor of one president, Philip H. Dubois, who was a pioneer in machine test scoring, and worked in areas such as test construction and validity (Dubois, 1970).

Herbert Woodrow wrote his dissertation in 1909 at Columbia University.^3^ His main interests were educational and experimental psychology. At the University of Illinois, he was the advisor of Hubert E. Brogden, who was president of the Psychometric Society in 1956. Most of Brogden’s contributions were in the fields of military psychology and personnel selection.

#### The Genealogy of Wilhelm Wundt, Part III

Wundt’s genealogy continues with four more graduate students (see Fig. 5). Edward Titchener wrote his dissertation in 1892 on binocular vision under Wundt’s supervision. After finishing his dissertation, he joined Cornell University where he developed a psychology laboratory. Unlike Cattell, Hall, and James, Titchener warmed up to Wundt’s methods. Similarly to Wundt, Titchener believed that psychology could be investigated through experiments, especially experimental introspection (Greenwood, 2015). Titchener advised one president of the Psychometric Society: Joy P. Guilford, who was the third president of the Psychometric Society in 1937. One of Guilford’s most famous studies involves the Structure of Intellect theory, in which intelligence could be traced back to a number of mental abilities (Guilford, 1956).

Hugo Münsterberg was a German psychologist, who first studied under Wundt and then joined William James at Harvard, where he became director of the psychology laboratory. His work was mostly applied, and he was a forerunner of professional psychology in the USA. Münsterberg was the advisor of Robert M. Yerkes, intelligence tester and eugenicist. Yerkes was famous for his research on the psychology and physiology of primates. During the First World War, he led the intelligence testing program, which resulted in the Army Alpha and Army Beta tests, for literate and illiterate recruits, respectively (Hilgard 1965). At Harvard University, Yerkes advised Melvin E. Haggerty, who started out as an animal researcher, but moved onto education later in his career. At the University of Minnesota, Haggerty was the advisor of Philip J. Rulon, president of the Psychometric Society in 1948. After finishing his dissertation in 1931, he joined Truman Kelley at Harvard University, and took to statistics and educational measurement.

Wundt’s fourth student in this genealogy is educational psychologist Charles H. Judd. He became director of the School of Education in 1909 at Yale University, and chairman of the Department of Psychology in 1920, following up James R. Angell (Buswell, 1947). From Judd, we found two lineages. The first one starts with Guy T. Buswell, an educational psychologist who became famous for his work on the psychophysiology of reading, and was a pioneer in the field of eye tracking. Buswell spent the first thirty years of his career at the University of Chicago, where Lee J. Cronbach was his graduate student. Lee Cronbach, president in 1953, is one of the most cited psychologists in the scientific literature (Haggbloom et al. 2002): his 1951 paper on the reliability coefficient alone has been cited over 35,000 times. He is also well known for his work on construct validity together with Paul Meehl (Cronbach & Meehl, 1955) and for developing generalizability theory (Cronbach, Rajaratnam, & Gleser, 1963).

The second student of Judd is the educational psychologist Adam R. Gilliland, doctoral advisor of Allen L. Edwards, who wrote his dissertation in 1940 and became president in 1963. Edwards was known for his skills for developing tests, and his most famous one was the Edwards Personal Preference Schedule, a personality inventory (Edwards, 1954).

With the exception of Wundt, we have so far only discussed psychologists and psychometricians who worked in the USA. One of the most influential psychometricians, however, was British: Charles Spearman (Fig. 5), who wrote his dissertation in 1906 with Wundt. He became famous for his work on intelligence and the notion of *general intelligence*, or *g*, which he modeled with the first latent variable model: the common factor model (Spearman, 1904). His work on reliability and its relation to test length (the Spearman–Brown formula) marked the beginning of classical test theory (Traub, 1997). In this genealogy, Spearman has only one student in this genealogy, Karl Holzinger, who was the advisor of Chester W. Harris^4^ (president in 1966). Both were well known for their work in exploratory factor analysis.

### The Genealogy of William James

William James has become known as the forerunner of American psychology. Including himself, his genealogy holds 16 scholars, of whom five became presidents of the Psychometric Society. James originally studied medicine, and in 1869 he earned his M.D. degree. James found that he was more interested in psychology and philosophy than medicine, but he never wrote a dissertation. It is well known that Wilhelm Wundt and William James diverged in their views on what kind of science psychology should be. Though James approved of Wundt’s experimental method and certainly learned from it, he also found it boring and unnecessarily rigorous (Hilgard, 1987). So like James M. Cattell, Hugo Münsterberg and Charles H. Judd, James returned to the USA without the intention of continuing the European experimental tradition.

Instead, William James became famous for his pragmatism, a quintessentially American philosophy. According to pragmatism, theories should be primarily evaluated by their practical usefulness, and the pragmatic method can be used for ‘settling metaphysical disputes that otherwise might be interminable’ (p. 28, James, 1975). Rather than formulating *psychological laws* that describe what human responses take place when a certain stimulus is shown, like Wundt aimed to do, James focused on the *function* of psychological concepts; their purposes in life. Wilhelm Wundt, in turn, was disillusioned by the pragmatism of his American students and thought that American psychology only focused on ‘inventions and physical comforts, rather than the deepening of man’s understanding of himself and of nature’ (Blumenthal, 1977, p. 18). This conflict foreshadows the tension between research traditions that emphasize pragmatic usefulness versus those that emphasize scientific understanding, which is present in many fields, including psychometrics—an example is the discussion of interpretation of psychometric models as merely being a useful description of the data versus scientific explanatory models of test behavior (e.g., see De Boeck & Wilson, 2004).

William James stayed at Harvard University, until he retired in 1907. Two of his students can be found in this genealogy: Granville Stanley Hall and Morris R. Cohen. In 1878, it was G. Stanley Hall who was the first to obtain a PhD degree in psychology awarded by an American university, under James’ supervision. Hall also spent a short time at Wundt’s laboratory, and like many of Wundt’s other students, Hall did not carry over many traditional German ideas on how to do psychological research. Hall established the first American experimental psychology laboratory at Johns Hopkins University and was initiator of the American Psychological Association in 1892. G. Stanley Hall was a pioneer in the field of educational psychology and believed that psychological research should serve a practical purpose and be applied to real-world problems.

From G. Stanley Hall, we found two separate lineages. The first one starts with Joseph Jastrow. In 1888, only two years after finishing his dissertation on spatial perception in vision and touch at Johns Hopkins University, he set up a psychological laboratory at the University of Wisconsin (Hull, 1944). During his career, Jastrow actively sought out the attention of the public, and wrote many popular publications besides his scientific work. One of Jastrow’s students at Wisconsin University was Clark L. Hull, known for the reinforcement theory of learning. Hull was a strong quantitative thinker and in all stages of his research, he strove for a certain mathematical rigor and solid theories.

Upon James Angell’s request, Hull took a position at Yale University in 1929. There, he headed a group of researchers who developed a research program devoted to the principles of learning, using rat experiments (Greenwood, 2015). Two of his graduate students are in this genealogy, Neal E. Miller and Carl I. Hovland (Fig. 6). Initially, Miller made an effort to experiment on Freudian ideas, like fear or frustration, and argued that behaviorist notions like conditional learning also applied to those. In the 1960s, he also took up brain studies, and became one of the biggest names in the new interdisciplinary field of ‘biofeedback.’ Neal E. Miller was the advisor of Gordon Bower, now emeritus cognitive psychologist. Bower left Yale University after finishing his dissertation in 1959 and went to Stanford University. For his dissertation, Bower used animal experiments to investigate reinforcement mechanisms, but when he left Yale, he concentrated more on mathematical models for memory. Bower was the advisor of Phipps Arabie, who was president of the Psychometric Society in 1990. Arabie was active in the fields of multidimensional scaling, clustering and combinatorial data analysis (Hubert et al., 2001). He became founding editor and guiding spirit of the *Journal of Classification*, the flagship journal of the Classification Society of North America (Hubert, 2012).

Another student of Hull in this genealogy was Carl I. Hovland. Hovland, who wrote his dissertation in 1936, always showed a wide interest in many topics and applied the experimental method to all sorts of problems. One of his best-known research projects took place during WWII, when he investigated the effectiveness of information programs on the motivation of American soldiers (Sears, 1961). Hovland was the advisor of Roger N. Shepard, president of the Psychometric Society in 1973. Shepard was a cognitive psychologist who belongs to the list of the 100 most eminent psychologists of the twentieth century (Haggbloom, 2002), especially because of his work on spatial relations and mental rotation. That work also led him to consider nonmetric approaches to multidimensional scaling, which inspired later presidents like Joe Kruskal, Forrest Young, Jim Ramsay, Yoshio Takane and Jan de Leeuw in their groundbreaking nonmetric approaches to multidimensional data analysis.

So far we have discussed the first lineage that starts with G. Stanley Hall at Johns Hopkins University. In 1888, G. Stanley Hall left Johns Hopkins University and became the first president of Clark University, a new university mostly dedicated to graduate education in psychology. There, he became the doctoral advisor of Lewis M. Terman, who wrote a dissertation on testing methods in 1905, even though Hall discouraged him from doing so (Hilgard, 1987). From 1922 until 1942, he was a professor of psychology at Stanford University, where he worked on what would become his most famous work: the Stanford Binet and the IQ test. One of his PhD students was Quinn McNemar, who was president in 1950. McNemar developed a revision of the Stanford-Binet Scale in 1942 and wrote his famous book *Psychological Statistics* (1949).

A second student of William James was Morris R. Cohen, who was prominent in a wide variety of domains, e.g., law, education and philosophy. His student, Ernest Nagel, became a famous philosopher of science. Nagel’s 1931 thesis was on the foundations of measurement, which continued to be an important theme in Nagel’s career. Other recurring topics were causality, scientific method and explanation, the foundations of probability and induction (Suppes, 1994). Ernest Nagel stayed at Columbia University for most of his career, where he was the advisor of Patrick Suppes, who finished his dissertation in 1950. Like his advisor, Suppes was a versatile scholar: he was well known as philosopher of science, but was also involved in experimental psychological research and founded a company that provided educational software (Moulines, 2016). After finishing his dissertation, he left for Stanford University where he advised many students, including two presidents of the Psychometric Society: Paul W. Holland and Larry Hubert. Paul W. Holland was active in many domains of the social sciences, e.g., educational testing at ETS, but also in social networks and causal inference. Larry Hubert is especially known for his work on graphs, trees and clustering. He was editor of *Psychometrika* from 1988 to 1992. In close collaboration with later presidents Phipps Arabie and Jacqueline Meulman, he developed the new field of combinatorial data analysis (Hubert, Arabie & Meulman, 2001).

### The Genealogy of Albert E. Michotte

Figure 7 displays the genealogy of Albert E. Michotte, a Belgian psychologist from Leuven University, who is the ancestor of four Dutch and two Belgian presidents. Though Albert E. Michotte also spent time with Wundt in Leipzig, Wundt was not his doctoral advisor. From Michotte, we found two pathways, one of which starts with the Dutch psychologist, Franciscus J. M. A. Roels who went to Utrecht University after finishing up his PhD degree in Leuven. He was well known for developing psychotechnic apparatus for the selection of employees. At Utrecht University, he was the advisor of F. J. Theo Rutten. Rutten became professor of psychology at Nijmegen University, and set up the psychology laboratory. His student, Alfons Chorus, was an assistant in that same laboratory. Both Rutten and Chorus believed that psychology could affect the world in a positive way and contribute to a more pleasant society. In 1947, Chorus became the founding professor of psychology at Leiden University.

In 1957, John van de Geer received his PhD degree under Chorus’ supervision. He was a cognitive psychologist who published one of the first sophisticated applications of nonmetric MDS (Levelt, Van de Geer & Plomp, 1996) and wrote an influential textbook about multivariate analysis (Van de Geer, 1971; cf. Heiser, 2008). He founded the highly regarded department of Data Theory in Leiden. Four of his students became president: Jan de Leeuw, Jacqueline Meulman, Willem Heiser, and Jos ten Berge (see Fig. 7). The first three integrated and extended the nonmetric techniques pioneered by Shepard, Kruskal and Guttman, publishing with their co-workers under the pseudonym Albert Gifi (cf. Van der Heijden & Sijtsma, 1996). Ten Berge became known for his work on reliability and factor rotation, but especially for his contributions to three-way data analysis, in the footsteps of presidents Tucker, Carroll and Kruskal.

The second branch that departs from Michotte starts with Joseph Nuttin, who, besides receiving his degree in 1941 from Michotte, was also an ordained priest. His main interests were learning, motivation and personality. He stayed in Leuven for his entire career, where he supervised Willem Claeys, a personality psychologist. Claeys was the advisor of Paul De Boeck, who is now professor of psychology at Ohio State University and was president of the Psychometric Society in 2007.

De Boeck’s work is characterized by a strong interest in individual differences and quantitative approaches, among which is explanatory item response models. Explanatory IRT exemplifies the idea that psychometric models are not merely technical vehicles for data analysis, but can also be used to build explanatory models of test behavior (De Boeck & Wilson, 2004). His student, Francis Tuerlincx, has become president of the Psychometric Society in 2018. Tuerlinckx’ work is strongly affiliated with mathematical psychology and combines important models taken from mathematical psychology (e.g., the diffusion model) with psychometric models (e.g., the IRT model; Tuerlinckx & De Boeck, 2005).

### The Genealogy of Carl Friedrich Gauss

The genealogies mentioned above have clear roots in psychology, and although the branches are separate when defined in terms of doctoral advisors of advisees, there are several occasions when people of separate genealogies met and even cooperated. However, not all presidents from the Psychometric Society have ancestors in psychology: an important subset of presidents, mostly of strong mathematical inclination, descends from the German mathematician Carl Friedrich Gauss. It is interesting to observe that two of the important ingredients of psychometrics—psychological research and mathematical analysis—are also visible in the form of distinct genealogies that can be traced back to psychology and mathematics. The genealogy of Gauss holds 40 scholars, including 9 presidents.

Carl Friedrich Gauss, being one of the most important mathematicians in history, contributed to a wide variety of fields, and Jones & Thissen (2007) consider his and Friedrich Wilhelm Bessel’s work (Bessel is a descendent of Gauss, see Fig. 8) work in astronomy as one of the cornerstones of psychometrics. He showed that the accumulation of small random deviations leads to a so-called Gaussian—or normal—distribution. The resulting ‘theory of errors,’ which involves the construction of a mathematical model that captures the behavior of measurement error, can also be considered the motivation for basic axioms in test theory (e.g., the notion that scores can be decomposed in a true score an error component as well as the idea that errors are uncorrelated and give rise to symmetric bell-shaped distributions; Lord & Novick, 1968). In addition, Bessel’s work was the first to give an analysis of differences between observers who recorded measurements of the exact same stimuli (i.e., positions of stars). Bessel developed the ‘personal equation,’ which corrected observations for systematic effects induced by different observers and in doing so gave one of the first formal approaches to the notion of *measurementbias*, which has also become a fundamentally important concept in test theory (Lord, 1980; Mellenbergh, 1989; Meredith, 1993).

As Fig. 8 shows, Gauss’ genealogy incorporates many mathematicians whose relevance to psychometrics is tangential. Describing each of them goes beyond the scope of this paper, so this section will only focus on the presidents who descended from Gauss.

Frederick Mosteller, president in 1957, and a student of Samuel S. Wilks, was a statistician with a profound interest in mathematical psychology, which resulted in a book *Stochastic Models for Learning* (1955), co-written with physicist Robert R. Bush. Over the course of his career, he collaborated with many prominent statisticians, among whom are Joseph B. Kruskal (see Fig. 5) and John Tukey (Fienberg, Hoaglin & Tanur, 2013). Kruskal, a student of Roger C. Lyndon and president in 1974, was a mathematician who, in the psychometric community, was most active in the domains of MDS and three-way data analysis. Besides his theoretical work, he also aimed to apply MDS to a variety of topics, like lexicostatistics or glottochronology (Carroll & Arabie, 2013). In 1958, he moved to Bell Labs in New Jersey, where he worked in research until his retirement.

William F. Stout, retired professor of statistics at the University of Illinois (where he stayed throughout his entire career), was president in 2001. Stout was a professor of mathematics for a number of years before he made the switch to statistics and psychometrics. In psychometrics, he made important contributions to Differential Item Functioning (DIF), unidimensionality and diagnostic classification. He was the doctoral advisor of three presidents of the Psychometric Society: Brian W. Junker, president in 2008, Hua-Hua Chang, president in 2012, and Daniel Bolt, current president-elect.

Melvin R. Novick, student of William J. Hall, was already mentioned as one of the two famous authors, the other being Fred Lord, of *Statistical Theories of Mental Testing* (1968). He remained active in the world of testing and became a frequent advisor at ETS. He was a pioneer of the use of Bayesian statistics in psychometrics (Lindley, 1987) and co-designed a statistical computing system, the CADA monitor (Novick, Hamer & Chen, 1979).

The last two presidents who connect to the Gauss genealogy are Karl G. Jöreskog and Bengt O. Muthén. Jöreskog was president in 1977 and became one of the most influential psychometricians of the second half of the twentieth century. After finishing his dissertation in Uppsala in 1963, he left for ETS, working together with Frederick Lord and Melvin Novick (Wainer, 2011). He returned to Sweden in 1971 to become one of the forerunners of Structural Equation Modeling (SEM). Together with Dag Sörbom, he developed the statistical program LISREL—the first widely available psychometric software program to become a success throughout the sciences. Jöreskog supervised many PhD students, among whom was Bengt Muthén, who became president in 1988. Muthén is now professor emeritus at UCLA. He is one of the developers of the computer program *Mplus*, which is specialized in latent variable modeling and implements many important extensions of the structural equation model.

### Lineages of Other Presidents

Including the president-elect of 2018–2019, there are 84 presidents of which 20 presidents cannot be connected to any of the genealogies above. In this section, we discuss their lineages, as represented in Table 1. The table shows the lineages for each of these presidents separately, including the year they wrote their dissertation, the university where they were a graduate student and their year of presidency.

Some of the presidents (e.g., Ivo W. Molenaar, R. Duncan Luce, Michael W. Browne) in this table have a lineage completely separate from other presidents. There are also a number of advisor–advisee couples in this table, for which one lineage holds two presidents. For example, Cees Glas, president in 2017, was the student of Willem van der Linden (president in 1999), Lyle V. Jones, president in 1962, was the advisee of Lloyd G. Humphreys (president in 1959), and Klaas Sijtsma, president in 2010, was the student of Ivo W. Molenaar, president in 1997.

**Table 1:** Year and University of Graduation, Year of Presidency and Lineages of ‘Unconnected’ Presidents of the Psychometric Society.

Name of president	Year of graduation	University where dissertation was written	Year of presidency	Lineage
Cees Glas	1989	University of Twente	2017	Wim J. van der Linden–Gideon J. Mellenbergh–Adriaan D. de Groot–Géza Révész–Georg E. Müller–Hermann Lotze
Sophia Rabe-Hesketh	1992	King’s College, University of London	2014	James F. Boyce
Anders Skrondal	1996	University of Oslo	2013	Petter Laake
Mark R. Wilson	1984	University of Chicago	2011	Benjamin D. Wright–Bruno Bettelheim–Robert Reininger
Klaas Sijtsma	1988	University of Groningen	2010	Ivo W. Molenaar–Jan Hemelrijk–David van Dantzig–Bartel L. van der Waerden–Hendrick de Vries–Diederik J. Korteweg
Wim J. van der Linden	1980	University of Amsterdam	1999	Gideon J. Mellenbergh–Adriaan D. de Groot–Géza Révész–Georg E. Müller–Hermann Lotze
Susan E. Embretson	1973	University of Minnesota	1998	Rene Dawis–Donald G Paterson
Ivo W. Molenaar*	1970	University of Amsterdam	1997	Jan Hemelrijk–David van Dantzig–Bartel L. van der Waerden–Hendrick de Vries–Diederik J. Korteweg
Fumiko Samejima	1965	Keio University	1996	Taro Indow
Gerhard H. Fischer	1963	Vienna University	1994	Erich Mittenecker–Hubert Rohracher–Erich Becher–Benno Erdmann
Michael W. Browne*	1968	University of South Africa	1991	Hendrik S. Steyn–Alexander C. Aitken–Edmund T. Whittaker–Andrew R. Forsyth–Andrew Cayley
Roderick P. McDonald	1963	University of New England	1985	John Keats
Peter M. Bentler	1964	Stanford University	1982	Douglas Jackson–John M. Hadley–John R. Knott–Lee E. Travis–Carl E. Seashore–George T. Ladd
R. Duncan Luce*	1950	Massachusetts Institute of Technology	1976	Irvin S. Cohen–Oscar A. Zariski–Guido Castelnuevo–Giuseppe Veronese–Antonio L. G. G. Cremona
Louis Guttman	1942	University of Minnesota	1970	F. Stuart Chapin–Franklin H. Giddings
Henry F. Kaiser	1956	University of California	1969	Harold D. Carter–Donald G. Paterson
Harry H. Harman			1968	Never wrote a dissertation
Lyle V. Jones	1950	Stanford University	1962	Lloyd G. Humphreys–Ernest Hilgard–Raymond Dodge–Benno Erdmann
John B. Carroll	1941	University of Minnesota	1960	B. F. Skinner–William J. Crozier–Jacques Loeb
Lloyd G. Humphreys	1938	Stanford University	1959	Ernest Hilgard–Raymond Dodge–Benno Erdmann

*The entire lineages of Ivo W. Molenaar, Michael W. Browne and R. Duncan Luce can be found on the website of the Mathematics Genealogy Project; in this table we only mention five of their ancestors.

Surprisingly, the lineage of Jones and Humphreys, both American psychometricians, and the lineage of Gerhard Fischer, psychometrician from Vienna University, share a common ancestor: Benno Erdmann. Benno Erdmann was a German Neo-Kantian philosopher, who held positions at different of German universities. At Halle University, he advised Raymond Dodge, an American philosophy student who was not admitted to Harvard or Columbia and crossed the Atlantic instead (Miles, 1956). Dodge returned to the USA, and specialized in experimental psychology. At Yale University, he advised Ernest Hilgard, the advisor of Humphreys. The second branch from Erdmann goes via Erich Becher, Hubert Rohracher and Erich Mittenecker to Gerhard Fischer, a specialist in IRT, who became president in 1994.

The individual lineages of Susan Embretson, president in 1998, and Henry F. Kaiser, president in 1969, also share a common ancestor: Donald G. Paterson. He was a pioneer in applied psychology, especially in the field of vocational guidance. Though he studied with Rudolph Pintner, a former student of Wilhelm Wundt, he never officially wrote his dissertation.

Table 1 also shows that for a number of presidents (Sophia Rabe-Hesketh, Anders Skrondal, Fumiko Samejima and Roderick McDonald), we have only included their own advisor. The rest of their lineages are simply unknown to us. It is unlikely though that their lineages will connect to any of the other existing lineages, as they either come from different fields (Rabe-Hesketh has a background in physics, Skrondal in biostatistics) and/or have received their training in different countries that are not strongly represented in this genealogy (resp.: United Kingdom, Norway, Japan and Australia). Therefore, we have refrained from further research into their genealogies.

Louis Guttman, president in 1970, is an important outsider in this genealogy. He was born in New York, received his PhD training in quantitative sociology at the University of Minnesota with his advisor, F. Stuart Chapin, and graduated in 1942. He then immigrated to the new state of Israel in 1947, where he became the founding director of the Israel Institute of Applied Social Research. Guttman gained an outstanding reputation as a psychometrician, due to his contributions in the American Soldier project during the Second World War, his work on exploratory factor analysis and MDS, and most famously perhaps, to the deterministic scaling model that carries his name (Guttman, 1944).

Mark R. Wilson was the graduate student of Benjamin D. Wright, who was a famous psychometrician in the Rasch model tradition. Wilson was president in 2011 and collaborated with Paul De Boeck on explanatory item response theory (De Boeck & Wilson, 2004). Wilson’s focus lies with a wide variety of measurement and assessment issues and is now professor at the Graduate School of Education of UC Berkeley.

Peter M. Bentler, president in 1982, was the doctoral student of Canadian psychologist Douglas N. Jackson. Bentler, though trained as a clinical psychologist, is especially known for his work on SEM and the development of EQS, a software package for SEM modeling (Bentler, 1995). He has also contributed to the fields of personality and drug abuse.

John B. Carroll, president in 1960, wrote his dissertation under B. F. Skinner, the famous behaviorist, at the University of Minnesota, but during his time as a graduate student, spent time at Thurstone’s laboratory at the University of Chicago. Thurstone had a strong influence on him, perhaps even more than Skinner, and his dissertation was on factor analysis of verbal abilities. Throughout his career, he made important contributions to the fields of intelligence research and testing.

Harry Harman is the only president without a doctorate degree and is therefore without a lineage. However, he made many contributions to the field. In 1936 he obtained a Master’s degree from the University of Chicago, which at the time was the center of psychometric research (see Fig. 2). He closely collaborated with Karl Holzinger in many articles and co-wrote a handbook on factor analysis (Holzinger & Harman, 1941).

## Conclusion and Discussion

We have developed a systematic overview of the lines of descent of Psychometric Society presidents through the construction of an academic genealogy. The graphs provide a broad overview of the people involved in these lines of descent and how they are interconnected; in addition, the genealogy is the first of its kind that provides verifiable evidential backup for each of the connections in the system. The genealogies we have reported have first and foremost a strongly descriptive purpose, as they describe the sequence of people in several individual lines of descent and identify common ancestors. Since the historical research necessary to document the relevant relations becomes more complicated as time goes on, the current paper also has an important function of preserving and disseminating this historical knowledge.

Besides these descriptive and preservationist purposes, genealogies can also serve as stepping-stones for further hypothesizing about the history of psychometrics. In the following, we present hypotheses about the history of psychometrics that are derived from one or more of the graphs.

### Missing Offspring

One of the questions that this genealogy raises is why certain scholars have produced a number of presidents, whereas other scholars, some of whom were equally if not more prominent in psychometrics, have very little or no offspring at all in the genealogy. An interesting example of an important name in psychometrics that has not had that same productivity within the Psychometric Society is Lee J. Cronbach. Cronbach is probably one of the most well-known psychometricians and is certainly the best cited psychometrician in psychology at large (Ho & Hartley, 2016). It is therefore quite surprising that Cronbach is a terminal node in the genealogy presented here.

Another influential psychometrician who is not prominently present in the genealogy is Charles Spearman. Spearman is probably one of the most influential psychometricians that ever lived, and his work has marked the birth of psychometrics as a discipline. However, unlike Thurstone’s, Spearman’s lineage in this genealogy is rather short. So why is it that Thurstone’s ‘formal’ influence reaches so much further than Spearman’s? One of the reasons might be that it was Thurstone who formalized the discipline by setting up the Psychometric Society and *Psychometrika* in the USA. Thurstone set up his own laboratory, first in Chicago, and later on at the University of North Carolina, and collected a large group of people that shared his ideas and developed them further (see Fig. 1), but Spearman never officially connected to this group. And though Thurstone and his followers were greatly inspired by Spearman and shared an interest in measuring mental abilities, they disagreed on some fundamental points, one being the number of mental abilities. So not only was Spearman not part of the Chicago-group, his and Thurstone’s work were simply incompatible (Thompson, 1947).

A number of influential psychometricians are entirely missing from this genealogy, such as Gustav Fechner, Francis Galton, and Raymond B. Cattell. The genealogy does not give an integrative explanation why some psychometricians are missing from the genealogy, and others are only marginally present. There are simply too many factors that can play a role: geography, interpersonal conflict, involvement with other groups, or simply a lack of graduate students who were active within the Psychometric Society. What these examples do show is that the number of descendants does not have a one-to-one relation with the extent to which a psychometrician was successful or influential: but rather with the extent to which a psychometrician was influential in the *formalization *and the *institutionalization* of psychometrics as an independent scientific discipline, and specifically, in the institutionalization of the Psychometric Society. So interpreted, our genealogy shows that these two kinds of importance do not necessarily coincide. This may be an important finding in the light of the way psychometricians portray the history of their field; psychometrics as an institutionalized discipline is more the field of Thurstone, Gulliksen, and Bock (those that trained students) than that of Spearman and Cronbach.

### Disciplinary Boundaries

The results section shows that the majority of psychometric presidents can be divided into a number of genealogies rooted in psychology and one genealogy that is rooted in mathematics. These findings suggest that psychometrics is an inherently multidisciplinary field, which embodies distinct traditions coming out of mathematics (specifically, statistical analysis) and psychology (specifically, psychological measurement and educational testing). We suggest that this inherent multidisciplinarity is responsible for the considerable tension between substantive and technical orientations that has played an important role in the history of Psychometric Society.

This tension has been particularly visible in several splits that have occurred in the history of psychometrics and that have led to the formation of at least two distinct professional organizations that deal with topics that were originally seen as belonging to the realm of psychometrics (Green, 1986). First, during the history of psychometrics, a sharp line was drawn between psychometrics and mathematical psychology. In the 1960s, several mathematical psychologists such as Duncan Luce and Patrick Suppes founded the Society for Mathematical Psychology and its flagship journal, *The Journal of Mathematical Psychology*, thereby formally separating mathematical psychology from psychometrics. Ever since, the two have existed in distinct realms.

Another important and very similar schism took place with the formation of the Society for Experimental Psychology (SMEP) in 1960 and its journals *Multivariate Behavioral Research* and the less-known *Multivariate Experimental Clinical Research* in 1966. Although this event appears less clearly related to psychometrics as a discipline, in Cattell’s (1990, p. 49) recollection of the history of SMEP he does state that ‘members should be chosen on the basis of experimental, substantive work, not of virtuosity in statistical ideas,’ which appears to echo the concern that formalized psychological work received scant attention in psychometrics. Green (1986) in fact relates this event specifically to the editorial policy of *Psychometrika*, which was to only publish substantive work if it served the purpose of illustrating a larger psychometric theme and not for its own sake.

The lack of integration of psychology and psychometrics thus appears to be at least partly a problem of the Psychometric Society’s own making. Attempts to remedy this situation, for instance by including an Applications section in *Psychometrika*, have been only partly successful. The present study suggests that the tension between psychology and mathematics may in fact run deep: the Psychometric Society is in essence a combination of disciplines of different origins, which bring with them different investigative styles and research traditions. As a function of these repeated splits, however, psychometrics has been increasingly dominated by statistically oriented rather than psychological approaches, so much so that many psychometricians today see psychometrics as a branch of statistics rather than a branch that is ‘devoted to the development of psychology as a quantitative rational science’ (the original motto of *Psychometrika*, appearing on its masthead until 1984). This trend is in contrast to Thurstone’s (1937, p. 232) first presidential address to the society, in which he said ‘Let us remember that a psychological theory is not good simply because it is cleverly mathematical, that an experiment is not good just because it involves ingenious apparatus and that statistics are merely the means for checking theory with experiment. In the long run we shall be judged in terms of the significance, the fruitfulness and the self-consistency of the psychological principles that we discover.’

### Increasing Diversity

The Psychometric Society was originally an American institution, and the majority of the presidents have been North American and received training at one of the major universities there. The first president who did his graduate education outside of North America was Karl Jöreskog. The meeting he organized, in 1978 in Uppsala, Sweden, was also the first meeting of the Psychometric Society outside the USA or Canada. After Jöreskog’s presidency, many other non-Americans followed, and meetings in the Netherlands, Belgium, France, and many other countries have been organized since. In 2001 the first Asian meeting was organized in Osaka, and in 2019 the first South American IMPS will be held in Chile. A second increase in diversity that is evident in the history of the Psychometric Society relates to gender. We count four female presidents in the past 25 years (i.e., in the period 1993–2018), which does not seem like much. However, it represents a spectacular increase relative to the long period that went before (1935–1993), which counted only one female president (Dorothy Adkins, president in 1949). Clearly, there is an increase in diversity there as well. Interestingly, a similar process may operate at the level of substantive backgrounds of psychometricians, as judged by the increasing number of presidents that have received a different type of training from that of the early presidents: there is a sizable influx of presidents with a mathematics background, mostly in the later stages of the Psychometric Society’s history (see the Gauss genealogy, Fig. 8). This indicates that the development of psychometrics is characterized by an increasing diversity, both in terms of socio-cultural and gender composition and in terms of substantive background.

Both the increasing diversity, and the formation of the breakaway groups, also suggest that the Psychometric Society is now possibly less representative of the psychometric community than in its early days, when the psychometrics community was significantly smaller and centered around a small number of universities. Psychometrics has become a widely practiced discipline, and the Psychometric Society, though still of high importance for the psychometric community, no longer represents it entirely.

### Limitations

With the selection of the presidents of the Psychometric Society as the initial set of nodes in the genealogy come a number of limitations. By making a selection, we have excluded important or influential psychometricians that were never president. In fact, this genealogy only shows how ideas may be spread through advising students and looses sight of other ways psychometricians have been influential, such as being a productive author or a meeting organizer. We want to stress that we by no means intend to provide a complete account of the history of psychometrics, and that this genealogy only sheds light on parts of it. Nevertheless, this study offers an important contribution to the literature on the history of psychometrics. The presidents of the Psychometric Society have each fulfilled important roles in the development of psychometrics, and their lines of descent are thus of importance to the history of psychometrics. Further research is needed to do justice to the complexity of the history of psychometrics as a whole.

In line with the argument given above, groups that are not strongly affiliated with the Society might find little or no representation in this genealogy, despite the fact that they may have played important roles in other ways. Future research could try to uncover these groups by a co-author network analysis of publications in *Psychometrika* and in related journals like the *Journal of Educational and Behavioral Statistics*, *The Psychological Bulletin*, *Psychological Methods*, the *British Journal of Mathematical and Statistical Psychology*, *Multivariate Behavioral Research*, *Structural Equation Modeling* and *Applied Psychological Measurement*.

## Electronic supplementary material

Below is the link to the electronic supplementary material.

Supplementary material 1 (pdf 49 KB)

Supplementary material 2 (pdf 65 KB)

Supplementary material 3 (pdf 46 KB)

Supplementary material 4 (pdf 38 KB)

## Footnotes

1 As the source for the Gauss genealogy is the Mathematics Genealogy Project, we have excluded such a table from the Supplementary Material.

2 Important to note is that Henry Garrett was certainly not the only psychometrician who believed racial differences in intelligence were genetic. In the late nineteenth century and early twentieth century, many scientists were supportive of Eugenics, the movement which aimed at improving the genetic quality of human beings (Norrgard, 2008).

3 Woodrow’s dissertation mentions Cattell, Thorndike, Lee and Woodworth as professors that have supported him throughout his time as a doctoral student. He is mentioned here as Woodworth’s student, but his dissertation is not conclusive in that regard.

4 It is unclear whether Karl Holzinger was in fact Harris’ official advisor. Holzinger is mentioned in the preface of Harris’ dissertation as a main contributor, but so are Stephen M. Corey, Ralph W. Tyler, Paul B. Diederich and Osmond E. Palmer.
