# Supplementary material for: An Academic Genealogy of Psychometric Society Presidents
Source: Psychometrika. 2019 Jan 17;84(2):562–88. doi: 10.1007/s11336-018-09651-4 (PMC6502785; doi:10.1007/s11336-018-09651-4)
Supplement: Supplementary file 1 — Supplementary material 1 (pdf 49 KB) [file 11336_2018_9651_MOESM1_ESM.pdf]

Table 2

Evidential Sources for each Advisor-Student Relationship in the Angell Genealogy.

Name of Scholar	University of Graduation	Year of Graduation	Doctoral Advisor	Source
Harvey A. Carr	University of Chicago	1905	James R. Angell	W. S. Hunter (1951). *James Rowland Angell (1869-1949), A biographical memoir.* Washington, DC: National Academy of Sciences.
Louis L. Thurstone	Chicago University	1917	James R. Angell	Dissertation
Carl J. Warden	University of Chicago	1922	Harvey A. Carr	Personal communication with Library University of Chicago.
Paul Horst	Chicago University	1931	Louis L. Thurstone	Heiser, W., & Hubert, L. (2016). A Creation Narrative for the Psychometric Society and Psychometrika: In the Beginning There Was Paul Horst. *Psychometrika*, *81*(4), 1172-1176.
Harold O. Gulliksen	University of Chicago	1931	Louis L. Thurstone	Personal communication with Robert Cudeck.
Robert L. Thorndike	Columbia University	1935	Carl J. Warden	Dissertation
Clyde H. Coombs	University of Chicago	1940	Louis L. Thurstone	Poole, Keith. T. (2008). The evolving influence of psychometrics in political science. In J.M. Box-Sfeffensmeier, H. E. Brady & D. Collier (Eds.) *The Oxford Handbook of Political Methodology* (pp. 199-216). Oxford: University Press.

Ledyard R. Tucker	University of Chicago	1946	Louis L. Thurstone	Dorans, N. J. (2004). *A conversation with Ledyard R. Tucker.* ETS Publication.
Warren S. Torgerson	Princeton	1951	Harold O. Gulliksen	Torgerson, W. S. (1952). Multidimensional scaling: I. Theory and method. *Psychometrika, 17,* 401 – 419.
Bert F. Green, Jr.	Princeton University	1951	Harold O. Gulliksen	Dissertation
Frederic M. Lord	Princeton University	1951	Harold O. Gulliksen	Dissertation
Samuel J. Messick	Princeton University	1954	Harold O. Gulliksen	Dissertation
Norman Cliff	Princeton	1956	Harold O. Gulliksen	Personal communication with Robert Cudeck.
William M. Meredith	University of Washington	1958	Paul Horst	Dissertation
J. Douglas Carroll	Princeton University	1963	Harold O. Gulliksen	Dissertation
Robert Linn	University of Illinois	1965	Ledyard R. Tucker	Personal communication with Bill Stout.
Bruce Bloxom	University of Washington	1966	Paul Horst	Bloxom, B. (1967). Effects of anger-arousing instructions on personality questionnaire performance. *ETS Research Report Series, 1,* 1-14.
James O. Ramsay	Princeton University	1966	Harold O. Gulliksen	Personal communication with James Ramsay.

Forrest W. Young	University of Southern California	1967	Norman Cliff	Personal communication with Yoshio Takane.
Philip L. Smith	University of Illinois	1976	Robert Linn	Personal communication with Terry Ackerman.
Yoshio Takane	University of North Carolina at Chapel Hill and University of Tokyo	1977	Forrest W. Young	Personal communication with Yoshio Takane.
Robert Cudeck	University of Southern California	1980	Norman Cliff	Personal communication with Robert Cudeck.
Roger E. Millsap	University of California	1983	William M. Meredith	Maydeu-Olivares, A. (2014). In Memoriam, Roger E. Millsap 1954-2014. *Psychometrika 79,* 355-356.
Terry A. Ackerman	University of Wisconsin-Milwaukee	1984	Philip L. Smith	Personal communication with Terry Ackerman.
