# Supplementary material for: An Academic Genealogy of Psychometric Society Presidents
Source: Psychometrika. 2019 Jan 17;84(2):562–88. doi: 10.1007/s11336-018-09651-4 (PMC6502785; doi:10.1007/s11336-018-09651-4)
Supplement: Supplementary file 2 — Supplementary material 2 (pdf 65 KB) [file 11336_2018_9651_MOESM2_ESM.pdf]

Table 3

Evidential Sources for each Advisor-Student Relationship in the Wundt Genealogy.

Name of Scholar	University of Graduation	Year of Graduation	Doctoral Advisor	Source
Hugo Münsterberg	University of Leipzig	1885	Wilhelm Wundt	Hilgard, E. R. (1987). *Psychology in America: A historical survey.* Orlando, FL: Harcourt, Brace, Jovanovich.
James McKeen Cattell	Leipzig Univeristy	1886	Wilhelm Wundt	Hilgard, E. R. (1987). *Psychology in America: A historical survey.* Orlando, FL: Harcourt, Brace, Jovanovich.
Robert S. Woodworth	Columbia University	1889	James McKeen Cattell	Boring, M. D. & Boring E. G. (1948). Masters and pupils among the American psychologists. *The American Journal of Psychology, 61,* 527 – 534.
Edward B. Titchener	University of Leipzig	1892	Wilhelm Wundt	Hilgard, E. R. (1987). *Psychology in America: A historical survey*. Orlando, FL: Harcourt, Brace, Jovanovich.
Charles H. Judd	University of Leipzig	1896	Wilhelm Wundt	Buswell, G. T. (1947). Charles Hubbard Judd: 1873-1946. *The American Journal of Psychology, 60,* 135 – 137.
Edward L. Thorndike	Columbia University	1898	James McKeen Cattell	Hilgard, E. R. (1987). *Psychology in America: A historical survey.* Orlando, FL: Harcourt, Brace, Jovanovich.

Robert M. Yerkes	Harvard University	1902	Hugo Münsterberg	Hilgard, E. R. (1987). *Psychology in America: A historical survey.* Orlando, FL: Harcourt, Brace, Jovanovich.
Charles E. Spearman	Leipzig University	1906	Wilhelm Wundt	Williams, R. H., Zimmerman, D. W., Zumbo, B. D., & Ross, D. (2003). Charles Spearman: British Behavioral Scientist. *Human Nature Review, 3,* 114 – 118.
Herbert H. Woodrow	Columbia University	1909	Robert S. Woodworth	Dissertation
Melvin E. Haggerty	Harvard University	1910	Robert M. Yerkes	Haggerty, Melvin, E. (Melvin Everett), 1875-1937. (n.d.). Retrieved from http://snaccooperative.org/ark:/99166/w6f197qx
Albert T. Poffenberger	Columbia University	1912	Robert S. Woodworth	Schoenfeld, W. N. (1979). Albert Theodore Poffenberger: 1885 – 1977. *The American Journal of Psychology, 92,* 143 – 149.
Truman L. Kelley	Columbia University	1914	Edward L. Thorndike	Hubert, L. (2013). Truman Lee Kelley (1884-1961) [pdf]. Retrieved from http://cda.psych.uiuc.edu/kelley_beamer_talk.pdf
Guy T. Buswell	University of Chicago	1920	Charles H. Judd	California Digital Library: http://texts.cdlib.org/view?docId=hb5g50061q&doc.view=frames&chunk.id=div00019 ;
Herbert A. Toops	Columbia University	1921	Edward L. Thorndike	Koppes, L. L. (2014). *Historical perspectives in industrial and organizational psychology.* Mahwah, NJ: Erlbaum.

Henry E. Garrett	Columbia University	1922	Albert T. Poffenberger	Dissertation
Karl John Holzinger	University of Chicago	1922	Charles E. Spearman	Sternberg, R. J., & Grigorenko, E. L. (eds). (2002). *The general factor of intelligence: How general is it?* Mahwah, NJ: Erlbaum.
Adam R. Gilliland	University of Chicago	1922	Charles H. Judd	Annotated List of Ph.D. Dissertations in Reading, 1916 – 1969. Chicago University.
Joy P. Guilford	University of Illinois	1927	Edward Titchener	Brett, J. M., & Drasgow, F. (Eds.). (2002). *The psychology of work: Theoretically based empirical research.* London: Lawrence Erlbaum Associates.
Harold A. Edgerton	Ohio State University	1928	Herbert A. Toops	Edgerton, H. A. (n.d.). A career in industrial and measurement psychology. Retrieved from http://www.siop.org/presidents/Edgerton.aspx
Robert. J. Wherry	Ohio State University	1929	Herbert A. Toops	Bartlett, C. J. (1982). The legacy of Robert J. Wherry, Sr. (1904-1981). *The Industrial-Organizational Psychologist, 5,* 7.
Irving Lorge	Teachers College, Columbia	1930	Edward L Thorndike	Thorndike, R. L. (1961). Irving Lorge. *Psychometrika, 26,* 1-2.
Jack W. Dunlap	Columbia University	1931	Edward L. Thorndike	Benjamin, L. T. (n.d.). The Early Presidents of Division 14: 1945-1954. Retrieved from http://www.siop.org/tip/backissues/tipoct97/BENJAM~1.aspx

Edward E. Cureton	Teachers College, Columbia	1931	Edward L. Thorndike	Shrader, R. R. (1994). Edward E. (Ted) Cureton (1902-1992). *American Psychologist, 49,* 350.
Philip J. Rulon	University of Minnesota	1931	Melvin E. Haggerty	Dissertation
Philip H. Dubois	Columbia University	1932	Henry E. Garrett	Thumin, F. J. (2002). Philip Hunter Dubois (1903 – 1998). *American Psychologist, 57,* 368*.*
John C. Flanagan	Harvard University	1935	Truman L. Kelley	Hubert, L. (2013). Truman Lee Kelley (1884-1961) [pdf]. Retrieved from http://cda.psych.uiuc.edu/kelley_beamer_talk.pdf
Dorothy C. Adkins	Ohio State University	1937	Herbert A. Toops	Adkins, Dorothy Christina, 1912-1975. Retrieved from http://snaccooperative.org/ark:/99166/w6hx2jvc
Hubert E. Brogden	University of Illinois	1939	Herbert H. Woodrow	Dissertation
Lee J. Cronbach	University of Chicago	1940	Guy T. Buswell	Shavelson, L. J. (2009) *Lee J. Cronbach (1916-2001), A biographical memoir.* Washington, DC: National Academy of Sciences.
Allen E. Edwards	Northwestern University	1940	Adam R. Gilliland	Summaries of Doctoral Dissertations, Northwestern University.
Ernest A. Haggard	Harvard University	1946	Truman L. Kelley	Personal communication with Harvard University Archives

Chester W. Harris	University of Chicago	1946	Karl J. Holzinger	Dissertation
Ben J. Winer	Ohio State University	1951	Robert J. Wherry	*Distinguished Teaching of Quantitative Methods in Psychology Award* [PDF file]. *American Psychologist, 39,* 313 – 314. Retrieved from: https://www.psychometricsociety.org/sites/default/files/Ben_J_Winer_from_Purdue.pdf
R. Darrell Bock	University of Chicago	1952	Ernest A. Haggard	Personal communication with R. Darrell Bock
Shizuhiko Nishisato	University of Chicago	1965	R. Darrell Bock	CV R. Darrell Bock
David Thissen	University of Chicago	1976	R. Darrell Bock	CV R. Darrell Bock
Robert Mislevy	University of Chicago	1981	R. Darrell Bock	Personal communication with Robert Mislevy
Ulf Böckenholt	University of Chicago	1985	R. Darrell Bock	CV R. Darrell Bock
Albert Maydeu-Olivares	University of Illinois	1997	Ulf Böckenholt	Personal Communication with Albert Maydeu-Olivares.
