# Supplementary material for: An Academic Genealogy of Psychometric Society Presidents
Source: Psychometrika. 2019 Jan 17;84(2):562–88. doi: 10.1007/s11336-018-09651-4 (PMC6502785; doi:10.1007/s11336-018-09651-4)
Supplement: Supplementary file 3 — Supplementary material 3 (pdf 46 KB) [file 11336_2018_9651_MOESM3_ESM.pdf]

Table 4

Evidential Sources for each Advisor-Student Relationship in the James Genealogy.

Name of Scholar	University of Graduation	Year of Graduation	Doctoral Advisor	Source
G. Stanley Hall	Harvard University	1878	William James	Hilgard, E. R. (1987). *Psychology in America: A historical survey.* Orlando, FL: Harcourt, Brace, Jovanovich.
Joseph Jastrow	John Hopkins University	1886	G. Stanley Hall	History of psychology. Retrieved from: http://www.learner.org/series/discoveringpsychology/history/history_nonflash.html
Lewis M. Terman	Clark University	1905	G. Stanley Hall	Boring. E. G. (1959)*. Lewis Madison Terman (1877 – 1856), A biographical memoir.* Washington, DC: National Academy of Sciences.
Morris R. Cohen	Harvard University	1906	William James	Personal communication with Harvard University Library
Clark L. Hull	University of Wisconsin	1918	Joseph Jastrow	Rieber, R. (2012). *Encyclopedia of the history of psychological theories*. New York: Springer.
Ernest Nagel	Columbia University	1931	Morris R. Cohen	Mathematics Genealogy Project
Quinn McNemar	Stanford University	1932	Lewis M. Terman	Hastorf, A. H. (2004). *History: Illustrious from past to present.* Retrieved from: https://psychology.stanford.edu/about/history

Neal E. Miller	Yale University	1935	Clark L. Hull	Coons, E. E. (2014). *Neal E. Miller (1909-2002), A biographical memoir.* Washington, DC: National Academy of Sciences.
Carl I. Hovland	Yale University	1936	Clark L. Hull	Hurley, K. P., & Hogan, J. D. (2017, June). *Carl Iver Hovland: A model general psychologist. A spotlight on Past-Presidents of APA Div. 1.* Retrieved from: http://www.apadivisions.org/division-1/publications/newsletters/general/2017/06/hovland-profile.aspx
Patrick C. Suppes	Columbia University	1950	Ernest Nagel	Mathematics Genealogy Project
Roger N. Shepard	Yale University	1955	Carl I. Hovland	Shepard, R. N. (1998). Carl Iver Hovland June 12, 1912 – April 16, 1961. *Biographical memoirs. National Academy of Sciences, 73,* 231 – 261.
Gordon H. Bower	Yale University	1959	Neal E. Miller	Chamberlin J. (2007). Psychologist wins National Medal of Science. *Monitor on Psychology, 38,* 10.
Paul W. Holland	Stanford University	1966	Patrick C. Suppes	Personal Communication with Paul W. Holland
Lawrence J. Hubert	Stanford University	1971	Patrick C. Suppes	Personal Communication with Larry Hubert
Phipps Arabie	Stanford University	1974	Gordon H. Bower	Personal communication Willem J. Heiser
