# Supplementary material for: An Academic Genealogy of Psychometric Society Presidents
Source: Psychometrika. 2019 Jan 17;84(2):562–88. doi: 10.1007/s11336-018-09651-4 (PMC6502785; doi:10.1007/s11336-018-09651-4)
Supplement: Supplementary file 4 — Supplementary material 4 (pdf 38 KB) [file 11336_2018_9651_MOESM4_ESM.pdf]

Table 5

Evidential Sources for each Advisor-Student Relationship in the Michotte Genealogy.

Name of Scholar	University of Graduation	Year of Graduation	Doctoral Advisor	Source
Franciscus J. M.A. Roels	Université Catholique de Louvain	1914	Albert E. Michotte	Busato, V., Essen, M. van., & Koops, W. (Eds.)(2013). *Vier grondleggers van de psychologie*. Amsterdam: Bert Bakker.
F.J. Theo Rutten	Utrecht University	1929	Franciscus J. M. A. Roels	Busato, V., Essen, M. van., & Koops, W. (Eds.)(2016). *Zeven grondleggers van de psychologie*. Amsterdam: Bert Bakker.
Alfons M. J. Chorus	Katholieke Universiteit Nijmegen	1940	F.J. Theo Rutten	Busato, V., Essen, M. van., & Koops, W. (Eds.)(2016). *Zeven grondleggers van de psychologie*. Amsterdam: Bert Bakker.
Jozef R. Nuttin	Catholic University Leuven	1941	Albert E. Michotte	Avermaet, E. van. (n.d.). JR Nuttin: Biography. Retrieved from https://ppw.kuleuven.be/home/english/faculty/jrnuttin/biography
John P. van de Geer	Leiden University	1957	Alphons M.J. Chorus	Busato, V., Essen, M. van., & Koops, W. (Eds.)(2016). *Zeven grondleggers van de psychologie*. Amsterdam: Bert Bakker.
Willem Claeys	Catholic University Leuven	1963	Jozef R. Nuttin	Personal communication with Paul De Boeck
Jan de Leeuw	Leiden University	1973	John P. van de Geer	Busato, V., Essen, M. van., & Koops, W. (Eds.)(2016). *Zeven grondleggers van de psychologie*. Amsterdam: Bert Bakker.
Paul De Boeck	Catholic University Leuven	1977	Willem Claeys	Personal communication with Paul De Boeck

Jos M. F. ten Berge	University of Groningen	1977	John P. van de Geer	Busato, V., Essen, M. van., & Koops, W. (Eds.)(2016). *Zeven grondleggers van de psychologie*. Amsterdam: Bert Bakker.
Willem J. Heiser	Leiden University	1981	John P. van de Geer	Busato, V., Essen, M. van., & Koops, W. (Eds.)(2016). *Zeven grondleggers van de psychologie*. Amsterdam: Bert Bakker.
Jacqueline J. Meulman	Leiden University	1986	John P. van de Geer	Busato, V., Essen, M. van., & Koops, W. (Eds.)(2016). *Zeven grondleggers van de psychologie*. Amsterdam: Bert Bakker.
Francis Tuerlinx	Catholic University Leuven	2000	Paul De Boeck	Personal communication with Francis Tuerlinckx
